# Automatic Adaptive Weld Seam Width Control Method for Long-Distance Pipeline Ring Welds

**DOI:** 10.3390/s25082483

**Published:** 2025-04-15

**Authors:** Yi Zhang, Shaojie Wu, Fangjie Cheng

**Affiliations:** 1 School of Materials Science and Engineering, Tianjin University, Tianjin 300350, China; zhangyi2021@tju.edu.cn (Y.Z.);; 2 China Petroleum Pipeline Research Institute Co., Ltd., Langfang 065000, China; 3 Tianjin Key Laboratory of Advanced Joining Technology, Tianjin 300350, China

**Keywords:** automatic welding, second-order derivative zero method, image processing, adaptive torch swing width tracking, laser sensor

## Abstract

In pipeline all-position welding processes, laser scanning provides critical geometric data of width-changing bevel morphology for welding torch swing control, yet conventional second-order derivative zero methods often yield pseudo-inflection points in practical applications. To address this, a third-order derivative weighted average threshold algorithm was developed, integrating image denoising, enhancement, and segmentation pre-processing with cubic spline fitting for precise bevel contour reconstruction. Bevel pixel points were captured by the laser sensor as inputs through the extracted second-order derivative eigenvalues to derive third-order derivative features, applying weighted threshold discrimination to accurately identify inflection points. Dual-angle sensors were implemented to synchronize laser-detected bevel geometry with real-time torch swing adjustments. Experimental results demonstrate that the system achieves a steady-state error of only 1.645% at the maximum swing width, a dynamic response time below 50 ms, and torch center trajectory tracking errors strictly constrained within ±0.1 mm. Compared to conventional methods, the proposed algorithm improves dynamic performance by 20.6% and exhibits unique adaptability to narrow-gap V-grooves. The results of these studies confirmed the ability of the method to provide real-time, accurate control for variable-width weld tracking, forming a swing-width adaptive control system.

## 1. Introduction

Laser scanning has recently matured and become a popular weld-tracking technology. The second-order derivative zero method is a classical edge detection technique that identifies the edge position in an image, but it often misjudges the edge position when dealing with non-linear, short, and thin lines, high noise, and poor clarity [1]. Researchers have enhanced the stability of identification using various improvement or transformation algorithms.

Recent innovations in multi-sensor fusion and deep learning have further expanded the capabilities of laser weld tracking. For instance, Ma et al. [2] proposed a transformer-optimized network for sub-millimeter defect detection in high-noise environments, achieving a 15% improvement in edge localization accuracy compared to conventional methods by integrating attention mechanisms with laser triangulation. Similarly, Yuan et al. [3] leveraged a DDPM-Swin Transformer hybrid to synthetically expand defect datasets, addressing data scarcity in welding inspections while maintaining a 0.1 mm resolution for groove geometry reconstruction. These approaches highlight the growing role of physics-informed AI in welding automation. In parallel, adaptive control strategies have emerged to address dynamic welding conditions. Zhang et al. [4] introduced an adaptive pseudoinverse control framework for hysteresis compensation in dielectric elastomer actuators, demonstrating a 30% reduction in steady-state error for non-linear systems—a methodology with potential applications in real-time torch positioning. Complementarily, Liu et al. [5] developed a constant-focus optical path system for thick-plate welding, achieving ±0.15 mm tracking accuracy through laser-ultrasonic fusion, though limited to fixed groove geometries. Wu et al. [6] proposed a laser stripe edge-guided network for autonomous seam recognition and feature extraction in multi-pass welding, integrating visual perception and deep learning to achieve real-time high-precision weld morphology detection and adaptive process parameter optimization. Gao, J. et al. [7] proposed a variable-gap weld feature extraction method that used the reflective properties of laser stripes in a fillet weld. A robust weld feature extraction model was established, a column grey difference operator was proposed, and the random sampling consistency algorithm was optimized to achieve weld tracking for variable-gap fillet welds. Zhu, C. H. et al. [8] proposed a vision system integrating multi-light sensing, dynamic image processing, and laser centerline extraction to achieve high-precision real-time detection and adaptive control of complex weld seam geometries. Despite these advancements, critical gaps persist. Yang et al. [9] identified that deep learning denoising methods (e.g., CNN-based approaches) struggle with real-time performance (<100 ms latency) when processing high-resolution laser scans (2048 × 2048 px), limiting their adoption in high-speed welding scenarios. Traditional methods, including second-order derivative algorithms and hybrid frameworks, remain constrained by pseudo-inflection point misidentification in variable-width grooves (e.g., V-type narrow gaps) under industrial noise levels (>60 dB). Sun, B. et al. [10] proposed a combinational structured light vision sensor that used the linear equation of the laser centerline and extracted feature points by solving the intersection through the joint equation. Zhang, G. et al. [11] proposed a second-order derivative algorithm to initially precisely position the feature points through linear fitting. Liu, X. et al. [12] proposed a laser-MIG composite weld seam width detection method based on a BP neural network compensated Kalman filter. The filter used the second-order difference method to obtain the weld seam contour width, while the BP neural network compensated for the optimal estimation of the Kalman filter. Ren, J. et al. [13] proposed a weld seam detection method based on the second-order anisotropic Gaussian directional derivative filter to improve key points in the image detection accuracy. Zhang, W. et al. [14] proposed a corner detection method using second-order generalized Gaussian directional derivatives, enhancing accuracy and noise robustness in complex scenes through optimized mathematical modeling. Cross-domain innovations bridge critical gaps in laser weld tracking.

Although laser-based weld seam tracking technology has advanced significantly, existing methods—including second-order derivative algorithms and hybrid deep learning frameworks—still suffer from limited robustness in high-noise environments and insufficient adaptability to variable geometric morphologies. This paper proposes an inflection point detection algorithm based on a third-order derivative weighted average threshold calculation, aiming to enhance the adaptability of laser tracking algorithms in complex engineering environments. This paper is structured as follows: Section 1 analyzes the advances and challenges in laser weld tracking. Section 2 describes the experimental setup, image pre-processing, and third-order derivative inflection point detection. Section 3 validates the laser sensing adaptive pendulum control, analyzes the dynamic response and accuracy, and discusses the applicability of the method. Section 4 summarizes algorithm advantages and applications.

## 2. System and Methods

### 2.1. System Composition

The experimental material was X80 pipeline steel of the main pipeline with a diameter of Φ1219 mm and a wall thickness of 30 mm. The welding consumables were an SG8-P (BOHLER Company, Kapfenberg, Austria), Φ1.0 mm solid wire. A V-type narrow gap bevel was used.

**Figure 1:**
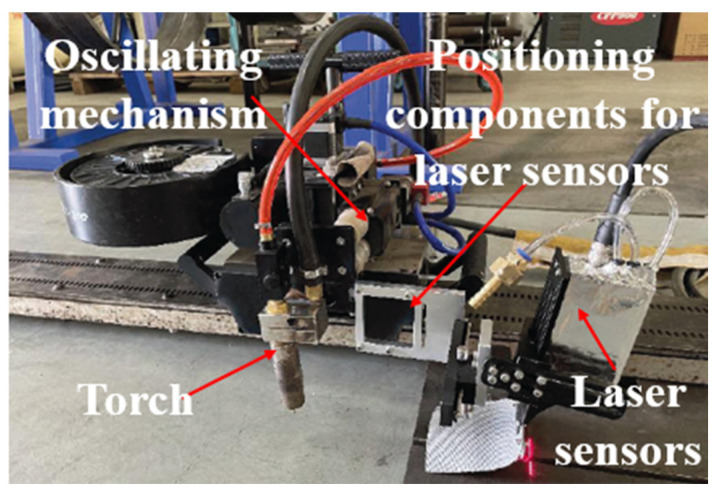
System configuration diagram.

The experimental equipment was a CPP900-W1N single torch external welding machine and laser sensor developed by the China Petroleum Pipeline Bureau. This system used the laser sensor front method, and it collected the weld morphology data by the laser triangulation principle and adjusted the relative X/Y/Z position and torch by the laser sensor holder. It welded the trolley assembly as shown in Figure 1.

The ring seam welding test was performed in accordance with the GB/T 31032-2023 [15] steel pipeline welding and acceptance standard. Gamma correction, non-linear median filtering, and the Canny algorithm were used for denoising, enhancement, and segmentation of the bevel image. The cubic spline algorithm was used to fit the image curve to obtain information about the bevel image points to extract the third-order function eigenvalues from the second-order function features. This was also used to obtain the discriminating thresholds of the image inflection points through the weighted average algorithm to accurately extract the location of the inflection points of the bevel and design weld seams with different widths. Welding experiments were used to verify the performance of the algorithm.

### 2.2. Pre-Processing Algorithm

Gamma correction was performed according to Equation (1):

(1) $$O=(2^{n}-1)\times {\left(\frac{I}{2^{n}-1}\right)}^{\gamma }$$

where O denotes the output pixel value of the image, I denotes the original pixel value at each point of the image, and γ is a non-linear parameter describing the relationship between the input and output. Greyscale details were enhanced by different values of γ. In different bit depths of the image, pixels were usually between 0 and $2^{n}$.

The original pixel I was normalized and restored to the bitmap after gamma correction. Based on the non-linear brightness perception of the human eye, the luminance response of the CRT monitor, and the sRGB standard, γ = 2.2 was selected [10].

A non-linear median filtering method was used to remove the image edges, and a 3 × 3 square window was used to enhance the image via Equation (2): [16]

(2) $$Y_{ij}=\sum_{3\times 3}Median\left\{x_{ij}\right\}$$

The Canny algorithm was used to segment the image into different regions, and a 3 × 3 Gaussian filter was selected to create a normalized Gaussian kernel matrix, expressed as Equation (3):

(3) $$H=\left[\begin{array}{ccc} 0.0573 & 0.1246 & 0.0573\\ 0.1246 & 0.2738 & 0.1246\\ 0.0573 & 0.1456 & 0.0573 \end{array}\right]$$

The Soble operator was used, and a 3 × 3 matrix was chosen as Equation (4):

(4) $$S_{x}=\left[\begin{array}{ccc} -1 & 0 & 1\\ -2 & 0 & 2\\ -1 & 0 & 1 \end{array}\right],\;\;\;S_{y}=\left[\begin{array}{ccc} 1 & 2 & 1\\ 0 & 0 & 0\\ -1 & -2 & -1 \end{array}\right]$$

The slope and direction were determined using Equation (5):

(5) $$\nabla f={\left\|\nabla f\right\|}_{2}=\sqrt{{G_{x}}^{2}+{G_{y}}^{2}}=\sqrt{{\left(\frac{\partial f}{\partial x}\right)}^{2}+{\left(\frac{\partial f}{\partial y}\right)}^{2}},\;\;\theta =arctan\left(\frac{G_{x}}{G_{y}}\right)$$

where G is the gradient magnitude, and θ is the gradient direction. Based on experience in suppressing noise while preserving edge information, the double threshold detection value was selected as follows: [14]

$$\;\frac{T_{h}}{T_{l}}=2.5$$

**Figure 2:**
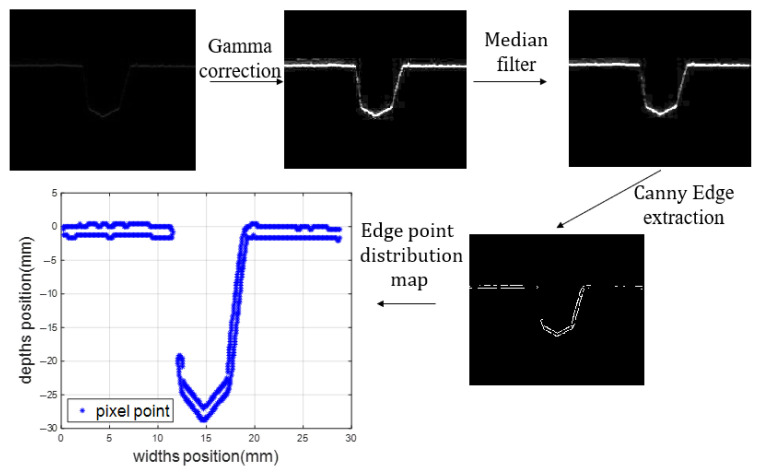
Pre-processing flow chart.

Figure 2 shows the flowchart for the bevel image pre-processing described above.

### 2.3. Third-Order Derivative Weighted-Average Threshold Inflection Detection Algorithm

The cubic spline function is expressed as Equation (6):

(6) $$s_{i}(x)=a_{i}+b_{i}\left(x-x_{i}\right)+c_{i}{\left(x-x_{i}\right)}^{2}+d_{i}{\left(x-x_{i}\right)}^{3}$$

Curve fitting was performed using a cubic spline, and diagonal matrices were constructed using the Thomas algorithm, as shown in Equation (7):

(7) $$\left[\begin{array}{ccccccccc} 1 & 0 & 0 & 0 & 0 & & & 0 & \\ {\Delta x}_{0} & 2\left({\Delta x}_{0}+{\Delta x}_{1}\right) & {\Delta x}_{1} & 0 & 0 & \cdots & & 0 & \\ 0 & {\Delta x}_{1} & 2\left({\Delta x}_{1}+{\Delta x}_{2}\right) & {\Delta x}_{2} & 0 & & & 0 & \\ & & \vdots & & & \ddots & & & \vdots \\ & & 0 & & & \cdots & {\Delta x}_{n-2} & 2\left({\Delta x}_{n-1}+{\Delta x}_{n-2}\right) & {\Delta x}_{n-1}\\ & & 0 & & & & 0 & 0 & 1 \end{array}\right]\cdot \left[\begin{array}{c} m_{0}\\ \begin{array}{c} m_{1}\\ \begin{array}{c} m_{2}\\ \begin{array}{c} \vdots \\ m_{n} \end{array} \end{array} \end{array} \end{array}\right]=6\left[\begin{array}{c} \begin{array}{c} 0\\ \frac{y_{2}-y_{1}}{{\Delta x}_{1}}-\frac{y_{1}-y_{0}}{{\Delta x}_{0}}\\ \frac{y_{3}-y_{2}}{{\Delta x}_{2}}-\frac{y_{2}-y_{1}}{{\Delta x}_{1}} \end{array}\\ \vdots \\ \begin{array}{c} \frac{y_{n}-y_{n-1}}{{\Delta x}_{n-1}}-\frac{y_{n-1}-y_{n-2}}{{\Delta x}_{n-2}}\\ 0 \end{array} \end{array}\right]$$

**Figure 3:**
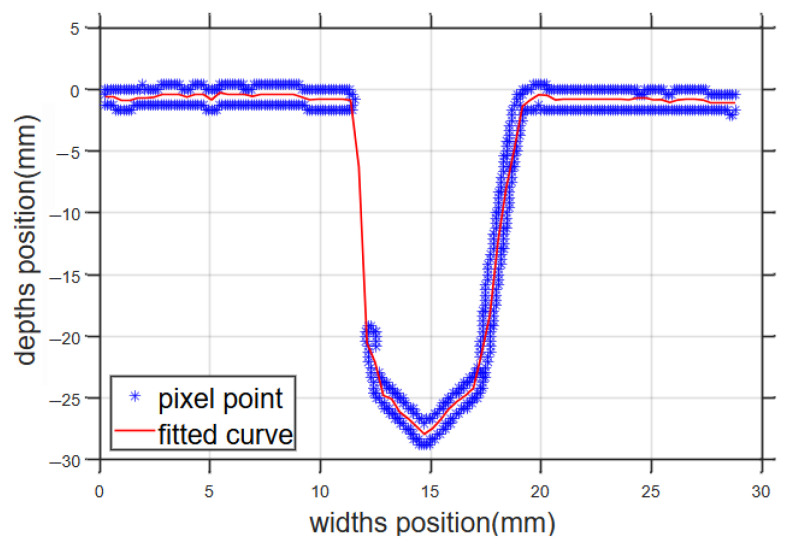
Bevel pixel position and fitted curve.

The system of diagonals was solved using Equation (8):

(8) $$m_{i}=\frac{6\left(\frac{y_{i+1}-y_{i}}{{\Delta x}_{i}}-\frac{y_{i}-y_{i-1}}{{\Delta x}_{i-1}}\right)-\frac{{\Delta x}_{i-1}}{c_{i-1}}d_{i-1}}{2\left({\Delta x}_{i-1}+{\Delta x}_{i}\right)-\frac{{\left({\Delta x}_{i-1}\right)}^{2}}{c_{i-1}}}$$

n intervals $m_{i},a_{i},b_{i},c_{i},d_{i}$ were solved, and the bevel profile was fitted, as shown in Figure 3.

The curve characteristics points were solved using Equation (9):

(9) $$\left\{\begin{array}{c} f^{\prime \prime }\left(x\right)=\frac{d}{d_{x}}\left(\frac{d}{d_{x}}\right)=0\\ f^{\prime \prime }\left(c-\delta \right)\cdot f^{\prime \prime }\left(c+\delta \right)<0 \end{array}\right.$$

**Figure 4:**
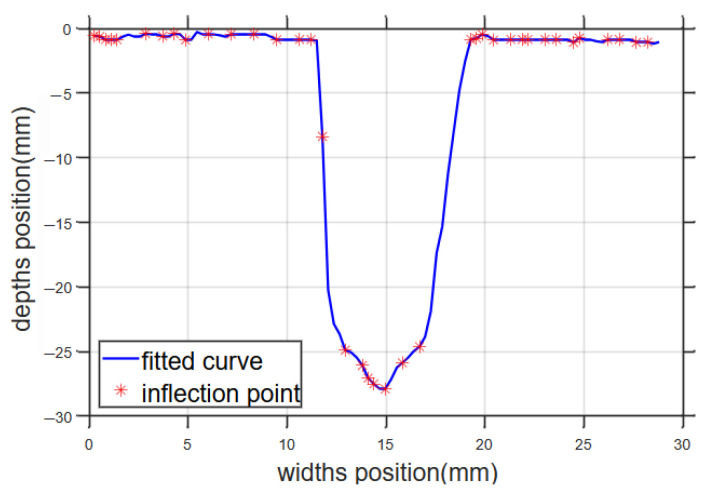
Cubic spline interpolation and inflection points.

Then, x=c is the point of inflection, and the inflection of the bevel fit image was plotted in Figure 4.

**Figure 5:**
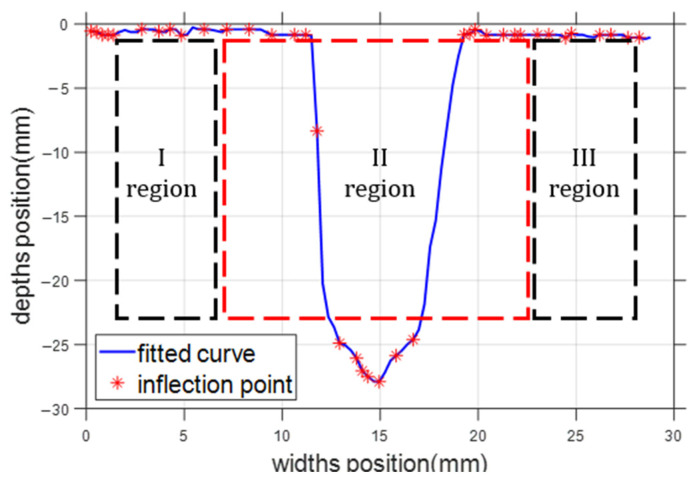
Division of fitted curve area.

Figure 4 contains multiple inflection points. The pseudo-inflection points were removed by extracting the third-order function eigenvalues from the second-order function features of the fitted curves, followed by calculating the weighted average, and finally, calculating the discriminative thresholds for the inflection points of the fitted curves. The image was divided into 3 regions, as shown in Figure 5.

The total number of pixel points was determined according to the pixel point data set $\left\{x_{1},\right.x_{2},x_{3},\cdots ,\left.x_{N}\right\}$, $\left\{y_{1},\right.y_{2},y_{3},\cdots ,\left.y_{N}\right\}$. The total number of pixel points in the curve fit was solved using Equation (10):

(10) $$n=\sum_{i=1}^{N}\delta (({x_{i},y}_{i}))\in P$$

where n is the total number of pixel points involved in the curve fitting, N is the size of the entire pixel point dataset, P is the number of pixel points used in the curve fitting, and δ is the indicator function (δ = 1 if the point $\left({x_{i},y}_{i}\right)$ belongs to the set P; otherwise, δ = 0). According to the width of the narrow gap bevel, we took the whole image lying on the center line to the left and right of the 40% of the pixel area that could cover all the pixel points of the narrow gap bevel. The region was divided into region I, region II, and region III, and the function was defined as Equation (11):

(11) $$R(x_{i},y_{i})=\left\{\begin{array}{c} Ι,\;\;\{({x_{i},y}_{i})|0\leq i\leq 0.2n\}\\ ΙΙ,\;\;\;\{({x_{i},y}_{i})|0.2n<i\leq 0.8n\}\\ ΙΙΙ,\;\;\{({x_{i},y}_{i})|0.8n<i\leq n\} \end{array}\right.$$

After the division, the region III weights were assigned a value of 2, and regions I and II were assigned a weight value of 1. The inflection point decrease was identified by the Eigen points of the third-order derivative function. The eigenvalues of the third-order derivative were solved using Equation (12):

(12) $$\dddot{s_{i}}\left(x\right)=\frac{\ddot{S_{i}}\left(x_{i+1}\right)-\ddot{S_{i}}\left(x_{i}\right)}{\left(x_{i+1}-x_{i}\right)}={6d}_{i}$$

where $s_{i}$ denotes the function of each segmented interval, and $x_{i}$ denotes the different interval data points. A weighted average was used to calculate the three regional averages using Equation (13):

(13) $$W=\frac{\sum_{i=1}^{n}w_{i}{\dddot{s_{i}}\left(x\right)}_{i}}{\sum_{i=1}^{n}w_{i}}$$

By introducing the defining function, the weight values were calculated using Equation (14):

(14) $$W=\frac{\sum_{i=0}^{0.2n}\dddot{s_{i}}\left(x\right)+2\sum_{i=0.2n+1}^{0.8n}\dddot{s_{i}}\left(x\right)+\sum_{i=0.8n+1}^{n}\dddot{s_{i}}\left(x\right)}{1.6n}$$

**Figure 6:**
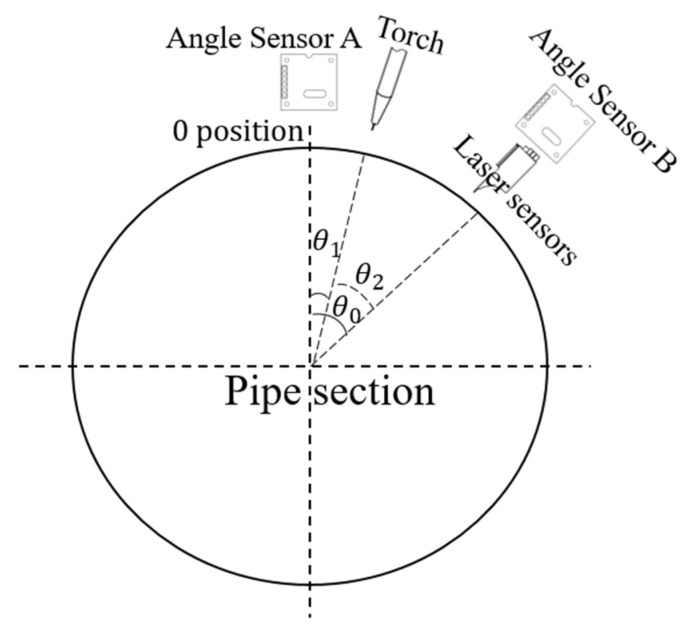
Inflection points identification relationship between position of angle sensor A, torch, and angle sensor B.

When ${\dddot{s_{i}}\left(x\right)}_{i}>W$, the inflection point was determined. The above algorithm was used to obtain the bevel cross-section data, and two angle sensors were used to establish the positional relationship between the laser sensor and oscillating torch, as shown in Figure 6. The figure shows that the angular relationship between the welding torch and the angle sensor B is ${\theta_{2}=\theta }_{0}-\theta_{1}$.

For different pipe diameters and travel speeds, the formula for calculating the number of images acquired per 1 degree is given as Equation (15):

(15) $$P_{i}=\frac{l\cdot f_{laser}}{v}=\frac{\pi \cdot D\cdot f_{laser}}{360\cdot v}$$

$P_{i}$ denotes the number of images acquired within a 1-degree range (i = 1, 2…), with the unit of “sheet”; l represents the arc length corresponding to 1 degree in millimeters (mm); D is the pipe diameter in millimeters (mm); $f_{laser}$ indicates the acquisition frequency of the laser sensor in hertz (Hz); and v is the travel speed of the welding carriage in millimeters per second (mm/s). These parameters collectively define the relationship between image acquisition and motion control during the welding process. The laser sensor’s frequency and the welding speed must be coordinated to ensure adequate image coverage and detection accuracy.

Using the D = 1219 mm pipe as an example, *l* = 10.6 mm, the welding speed of a normal welding process *v* = 300–600 mm/min, every 1° of running time was about 1.06–2.12 s, $f_{laser}$ = 10 Hz, every 1° of running collected 10–21 images, every 0.1° generated 1–2 images, and every 0.1° corresponded to a traveling distance of about 1 mm. The detection accuracy of the pipeline welding process meets usage requirements. The image reading angle was defined as Δ*θ* and was extracted once every 0.5°.

Before welding was started, the control system database was preset with a set of welding parameters for the annular weld, according to the position of 0–6 points, which were divided into 12 intervals. The resulting array was $S_{n}=\left\{1,2,\ldots 12\right\}$, each interval was 15° for the parameter preset, and the swing width array was written as ${SW}_{i}=\left\{{SW}_{1},{SW}_{2},\ldots {SW}_{12}\right\},i=(1,2,\ldots \frac{180}{15})$.

**Figure 7:**
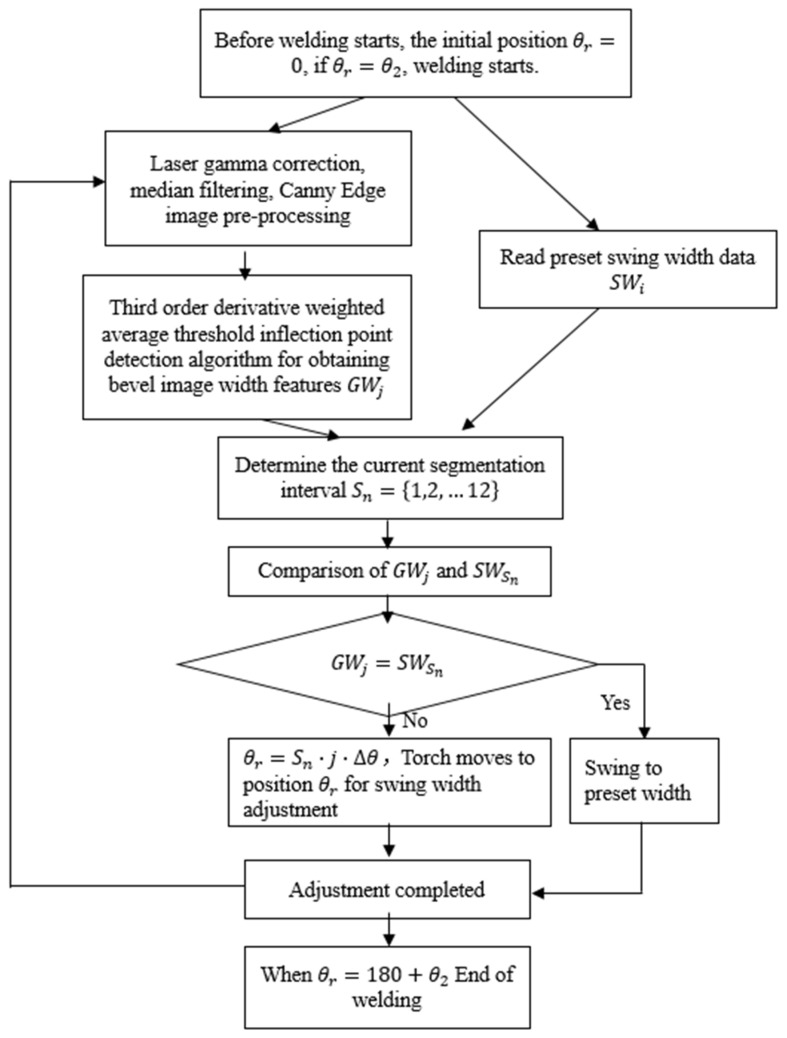
Adaptive swing width control flowchart.

The angle sensor B began to move with the welding carriage from the 0-point position, and the angle change of the angle sensor B was recorded as *θr*. Throughout the welding process, the two-dimensional image width feature of the bevel was extracted and calculated by the pre-processing of the laser sensor and the third-order derivative weighted average threshold detection algorithm, which was recorded as ${GW}_{j}$. According to the welding process parameter for every 15° interval, ${GW}_{j}$ was compared with the data of ${SW}_{i}$. According to the comparison result, the welding torch was automatically controlled to complete the swing width adaptive adjustment, and the flow chart is shown in Figure 7.

## 3. Results and Discussion

**Figure 8:**
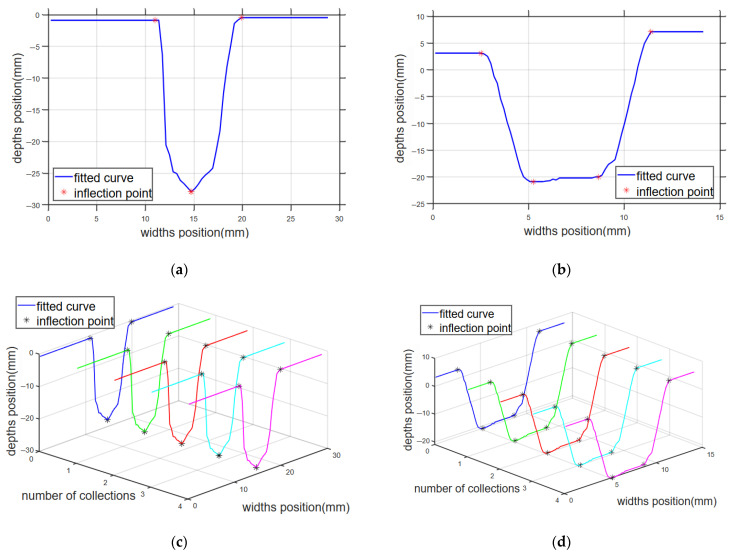
Inflection point identification and fitted curve; (**a**) cubic spline interpolation and inflection points of Groove1; (**b**) cubic spline interpolation and inflection points of Groove2; (**c**) Groove1 fitting curves and inflection points; (**d**) Groove2 fitting curves and inflection points.

Using the above algorithm, the weld bevel is scanned by the laser sensor front to calculate the bevel characteristic points, fitted bevel shapes, curves, and inflection points, as shown in Figure 8.

**Figure 9:**
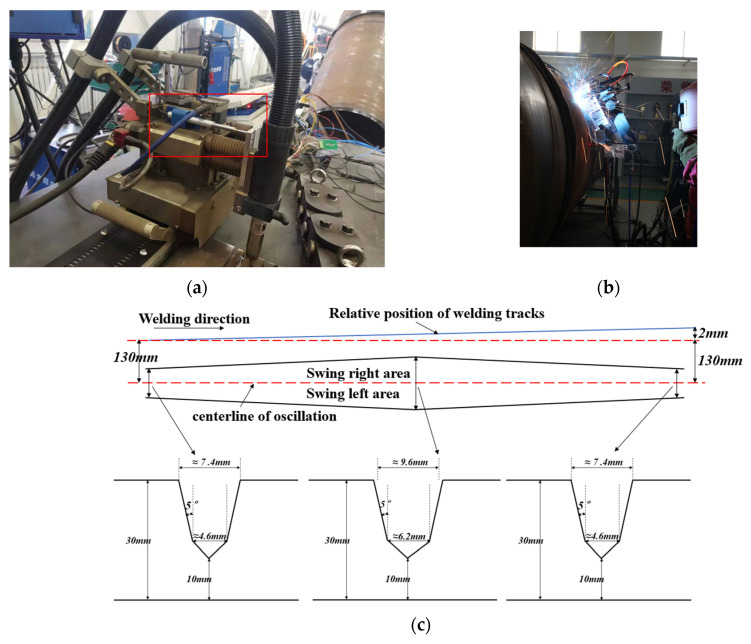
Experimental validation platform. (**a**–**c**) The diagram illustrates: a 5° narrow-gap groove is adopted for the joint preparation. The welding torch advances along the oscillation centerline, performing asymmetric amplitude oscillations of 7.4 mm, 9.6 mm, and 7.4 mm in groove width. The starting point of the welding track (left side) is 130 mm away from the oscillation centerline, while the end point (right side) shows a 2 mm positional deviation relative to the oscillation centerline.

In the experimental platform, the data acquisition system, using displacement sensors, was installed in the swing torch transverse cross-slide and was used to measure the position signal of the swing process to record relevant data. Using the swing width adaptive verification method, during the processing of a narrow-gap V-bevel, the upper and lower bevel surface angles were 5° and 45°, respectively. The lower opening width was 4.4 ± 0.2 mm, and the width of the bevel gradually expanded and then narrowed. The welding track and the torch centerline cumulatively deviated from the right side by 2 mm to observe the process of adjusting the welding torch. The overall schematic diagram is shown in Figure 9.

The parameters used for the welding process are shown in Table 1.

**Figure 10:**
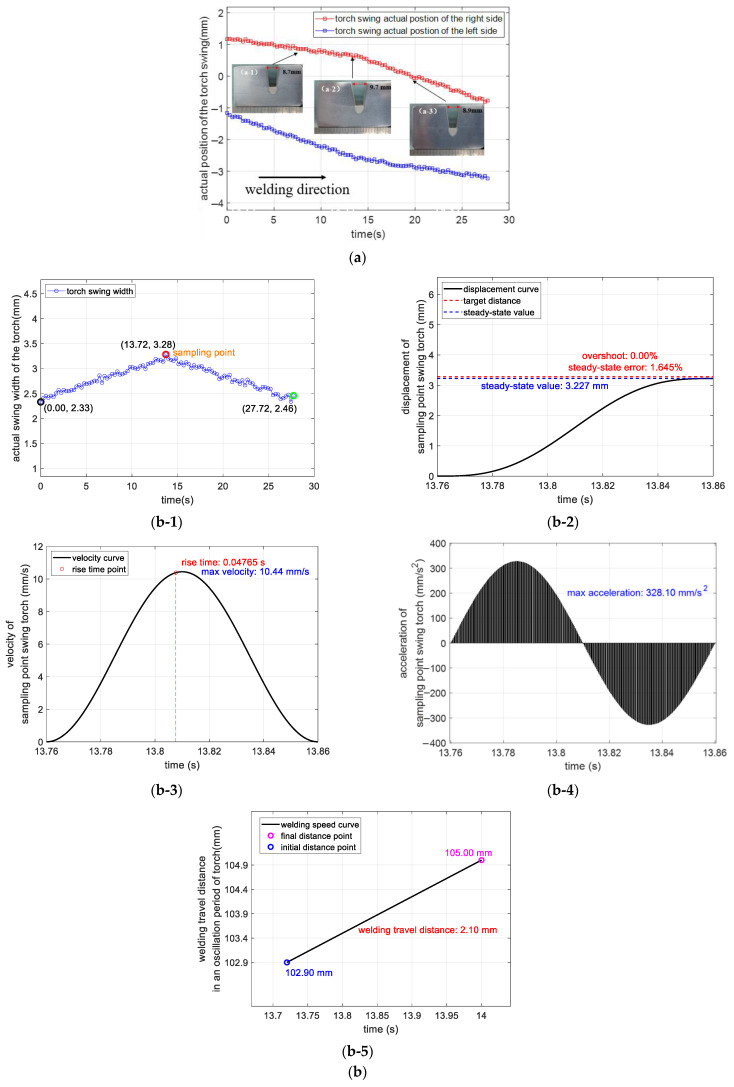

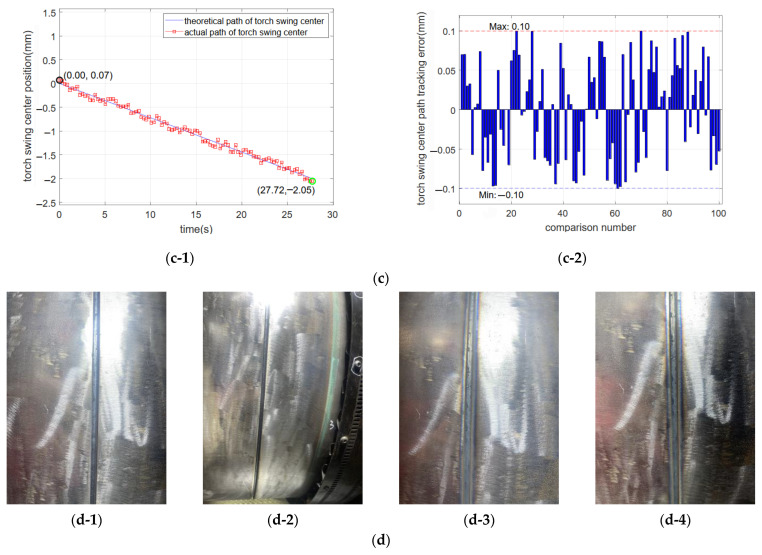
Torch swing width adaptive adjustment process; (**a**) left–right path comparison; (**b**) maximum oscillation sampling point performance chart; (**c**) torch centerline adjustment diagram; (**d**) weld seam shaping of different welding layers; (**b-1**) actual torch swing width; (**b-2**) sampling point torch adjustment displacement curve (mm); (**b-3**) sampling point torch adjustment velocity curve (mm/s); (**b-4**) sampling point torch adjustment acceleration curve (mm/s^2^); (**b-5**) sampling point welding travel distance in an oscillation period of torch curve; (**c-1**) center path comparison; (**c-2**) center position error; (**d-1**) first layer filler pass; (**d-2**) third layer filler pass; (**d-3**) fourth layer filler pass; (**d-4**) fifth layer filler pass.

The oscillation period of the torch was expressed as 2 × Oscillation time + 2 × Side stop time. After welding was started, the control system calculated the required torch swing parameters in real time according to the width data fed back from the laser sensor. The position signal from the displacement sensor was used to monitor the torch swing control parameter data. Three characteristic points were chosen to observe the weld shape in combination with the macroscopic metallographic image. The position signal of the displacement sensor was collected, the control parameter data of the torch swing was monitored, and three characteristic points were selected to observe the weld shape in combination with the macroscopic metallographic image. Change data of the torch swing width were used to determine the position of the widest width change of the bevel. The sampling point was designed to analyze the displacement, speed, and acceleration curves of the system controlling the torch swing process, as well as the welding path within the swing cycle. The change curves of the weld center position were collected and calculated, as well as the following error, as was the total welding path during a swing cycle. The variation curves of the weld center position were collected and calculated, as were the tracking error and total welding distance within the swing cycle, as shown in Figure 10.

Figure 10a shows the changes in the left and right trajectories of the torch, and the three marked points (Figure 10(a-1)–(a-3)) correspond to the widths of the top opening of the weld seam of 8.7 mm, 9.7 mm, and 8.9 mm, respectively. Figure 10b records the process during which the pendulum width increased from 2.33 mm to a maximum of 3.28 mm before recovering to 2.46 mm. It obtained the widest pendulum sampling point, and the measured pendulum distance was 3.28 mm. The displacement curve of the sampling point showed a steady-state error of 1.645% (steady state value of 3.227 mm), and the velocity curve had a rise time of 47.65 ms, a maximum velocity of 10.44 mm/s, and a peak acceleration of 328.1 mm/s^2^. The welding travel moved a distance of 2.1 mm in a single oscillation period of the torch. Figure 10c shows the adjustment of the torch center path and the statistics of the actual and theoretical center position errors, including the start point (0.00 s, 0.07 mm) to the endpoint (27.72 s, −2.05 mm), which showed a maximum error of 0.1 mm and a minimum of −0.1 mm.

The weld shape flatness and fusion quality showed that the system had an effective path-tracking capability. The continuous variation of swing width data confirmed that the adaptive algorithm responded to 1.4 times width fluctuations (2.33–3.28 mm) in real time. The steady-state error of the sampling point displacement was less than 2%, with no overshoot characteristics, verifying the stability of the control system. The velocity response time of 47.65 ms with a welding movement of 2.1 mm in one oscillation cycle indicated that the system achieved real-time tracking while maintaining a 0.3 m/s^2^ acceleration constraint. The central path deviation data matched the 2.0 mm deviation of the designed path, and the 0.2 mm peak-to-peak error band shows motion control accuracy.

The welding system achieved the tracking control of the variable width of the weld seam in terms of path tracking, with an adaptive adjustment range of the pendulum width of 40%, a dynamic response time of less than 50 ms, and a steady-state control accuracy higher than 98%. The motion control process had no overshoot characteristics, and the tracking error of the central path did not exceed ±0.1 mm, which demonstrates the reliability of the algorithm for the multi-parameter coupling control of velocity (10.44 mm/s), acceleration (328.1 mm/s^2^), and position (3.227 mm). This meets the real-time, accuracy, and stability requirements during complex weld seam formation.

Figure 10d shows the molding of different solder layers, including fillers 1, 3, 4, and 5. The weld bevels of different welding layers are well-shaped, indicating that based on the principle of third-order derivative weighted thresholding, combined with image pre-processing and real-time feedback control, the traditional method effectively solves the problem of being susceptible to noise interference and misjudging the edge position in complex welding scenes. Through real-time inflection point detection, the algorithm is able to quickly analyze the changes in the bevel shape and adjust the swing amplitude and path of the torch in real time, which improves the good adaptation of the torch to the changes in the width of the narrow-gap V-bevel. This thesis compares the performance with existing methods, as shown in Table 2.

As shown in Table 2, the proposed third-order derivative weighted threshold algorithm demonstrates performance across three critical dimensions: real-time capability, tracking accuracy, and adaptability. In terms of real-time performance, the dynamic response time of 47.65 ms outperforms state-of-the-art Z-groove anti-vibration methods [16] (60 ms) by 20.6%, primarily due to the elimination of multi-sensor synchronization delays and the optimized cubic spline fitting process. For tracking accuracy, the center trajectory error is constrained within ±0.1 mm, significantly surpassing lightweight models [20] (±0.4 mm) and matching the precision of 3D fusion methods [17] (±0.1 mm). This is attributed to the integration of dual-angle sensors and third-order derivative filtering, which effectively suppresses pseudo-inflection points in high-noise environments. Regarding adaptability, the algorithm uniquely supports V-type variable-width grooves without requiring preset parameters. The proposed method achieves the lowest tracking error (±0.1 mm) for V-type grooves while maintaining computational efficiency and real-time responsiveness.

Experimental results show that the proposed third-order derivative-weighted threshold algorithm achieves adaptive pendulum control under standardized industrial conditions for narrow-gap 5° V-grooves on X80 pipeline steel, particularly in filler layers with a tangible bevel feature. However, the applicability of this method is currently limited to cases involving precision machined grooves where beveling equipment ensures controlled geometry. Non-standard cases, such as flame-cut grooves or highly deviated manual assemblies, have not been validated due to irregular groove morphology and thermal deformation effects. A key limitation of the algorithm is its dependence on pre-processed groove geometry. While the dual-angle sensor system is effective in synchronizing the laser scan data with the torch adjustment under controlled conditions, in field applications involving heterogeneous materials or other groove profiles, it may be necessary to recalibrate the weighted threshold parameters and the cubic spline fitting criteria. For example, flame-cut grooves typically have high surface roughness and thermal distortion, which may introduce pseudo-inflection points even with third-order derivative filtering. To address these limitations, subsequent exploratory work should be continued. First, the modular structure of the core algorithm adds process-adaptive module integration, which compensates for the deformation effects of non-standard trenches. Second, collaborative trials with pipeline contractors will evaluate performance under real-world high-deviation conditions. Third, extending the validation to ISO standard geometries (e.g., U-grooves conforming to API 1104) and multiple grades of steel (X65–X100) will increase the versatility of the method. These steps are intended to transform the method from a lab-validated solution to a field-deployable tool while maintaining compliance with global welding standards. Furthermore, while the system demonstrates real-time capability, large-scale deployments may necessitate edge-AI optimizations to handle computational loads across extended welding operations. Future work will also explore cost–benefit trade-offs for retrofitting existing welding systems with the proposed laser triangulation and sensor modules, particularly in resource-constrained environments. To bridge these technical challenges with broader engineering innovations, recent advances in cross-domain methodologies could offer complementary insights. For instance, the multi-sensor fusion strategies in Collaborative Imaging of Subsurface Cavities Using Ground-Pipeline Penetrating Radar [23]—which integrates heterogeneous data for high-resolution defect detection—might inspire enhanced algorithms to mitigate thermal distortion in flame-cut grooves by combining laser triangulation with thermal imaging. Similarly, the microstructure-optimized approaches in Simulation of Ultrasonic Welding of Cu/Cu Joints with an Interlayer of Cu Nanoparticles [22], where nanoparticle-enabled interface refinement improves joint reliability, could inform future designs of adaptive filler materials for non-standard geometries. Additionally, the crack suppression mechanisms analyzed in Resistance Spot Welded NiTi Shape Memory Alloy to Ti6Al4V [24], which correlate welding parameters with residual stress distribution, may guide the development of stress-compensation modules for high-deviation scenarios.

## 4. Conclusions

This study proposes an inflection point detection algorithm based on a third-order derivative weighted average threshold. By extracting the eigenvalues of the third-order derivative function and combining them with weighted averaging of image regions, the algorithm automatically determines the inflection point discrimination threshold for groove images. Dual-angle sensors synchronize the laser-scanned groove geometry with real-time welding torch swing data, establishing an adaptive swing-width tracking control system. The effectiveness of the method was validated through welding experiments, leading to the following conclusions:

(1) This study integrates laser triangulation with a third-order derivative weighted threshold algorithm, addressing the limitations of traditional second-order derivative zero-crossing methods in generating pseudo-inflection points under noisy conditions. Through image pre-processing and cubic spline fitting, high-precision groove contour reconstruction was achieved. Combined with dual-angle sensor synchronization technology, the system significantly enhances real-time tracking capabilities for V weld seam geometries.
(2) Experimental results demonstrate that the system achieves a steady-state error of only 1.645% at the maximum swing width, a dynamic response time below 50 ms, and torch center trajectory tracking errors strictly constrained within ±0.1 mm. Compared to conventional methods, the proposed algorithm improves dynamic performance by 20.6% and exhibits unique adaptability to narrow-gap V-grooves.
(3) Multi-layer and multi-pass welding experiments confirmed uniform weld formation and excellent fusion quality, highlighting the algorithm’s practical potential for X80 pipeline steel narrow-gap girth welds. Compared to existing approaches, this method demonstrates superior tracking accuracy.

## Figures and Tables

**Table 1:** Table of relevant test parameters.

Number	Parameter	Value
1	Initial swing (mm)	2.4
2	Oscillation time (ms)	100
3	Side stop time (ms)	40
4	Travel speed (mm/min)	450.0

**Table 2:** Performance comparison between the method and representative studies.

Method/Reference	Method Type	Dynamic Response Time (ms)	Tracking Error (mm)	Adaptability to Grooves
Article Method	Third-Order Derivative + Weighted Threshold	47.65	±0.1	Narrow-Gap V
Ref. [9] (Deep Learning Denoising)	Deep Learning	N/A	±0.3	No
Ref. [17] (3D Tracking)	Laser + Vision Fusion	100	±0.1	Yes
Ref. [16] (Z-Groove Anti-Vibration)	Vibration Suppression	60	±0.2	Z-Groove
Ref. [18] (Multi-Sensor Fusion)	Laser + Robotic Control	75	±0.5	Partial
Ref. [19] (LSFP-Tracker)	Laser Stripe Feature Extraction	65	±0.3	Partial
Ref. [20] (Lightweight Model)	Knowledge Distillation + Pruning	50	±0.4	No
Ref. [21] (Feature Segmentation)	Additive Manufacturing Tracking	60	±0.5	Yes (Limited Width)
Ref. [22] (Laser Depth Measurement)	Laser Geometry Extraction	N/A	N/A	No

## Data Availability

All data generated or analyzed during this study are included in this published article.
